# Real-world assessment of the performance of EBUS-TBNA in diagnosing mediastinal lymph node metastases from extrapulmonary malignancies

**DOI:** 10.36416/1806-3756/e20250337

**Published:** 2026-11-09

**Authors:** Alejandro Golfe Bonmatí, Ana Núñez Ares, Raúl Godoy Mayoral, Marina Alcaraz Barcelona, Patricia López Miguel, Jesús Jiménez López

**Affiliations:** 1. Departamento de Neumología. Hospital Universitario de Albacete, Servicio de Salud de Castilla La Mancha - SESCAM - Albacete, España.; 2. Facultad de Medicina, Campus Albacete, Universidad de Castilla-La Mancha - UCLM - Albacete, España.

**Keywords:** Endosonography/instrumentation, Biopsy, fine-needle/instrumentation, Lymph nodes/pathology, Neoplasm metastasis/diagnostic imaging, Ultrasonography, Survival rate

## Abstract

**Objective:**

To evaluate the diagnostic performance and clinical implications of endobronchial ultrasound-guided transbronchial needle aspiration (EBUS-TBNA) for assessing mediastinal or hilar lymph node involvement in patients with extrathoracic malignancies (ETMs).

**Methods:**

This was a single-center, ambispective observational study including consecutive patients with an ETM and abnormal thoracic lymph nodes (LNs), detected by CT or PET/CT, who underwent EBUS-TBNA between January of 2012 and December of 2022. Diagnostic performance was assessed by using predefined criteria, with surgical confirmation or long-term clinical and radiological follow-up (for ≥ 24 months) as the reference standard.

**Results:**

We included 186 patients with a collective total of 237 LNs. The most common ETMs were colorectal, urological, and breast cancer (in 22.6%, 22.6%, and 20.4%, respectively). Most (72%) of the LNs sampled were mediastinal, predominantly subcarinal (in 33%) and right paratracheal (in 29%). We found that EBUS-TBNA had a sensitivity of 96.4% (95% CI: 90.98-98.62%), a negative predictive value of 94.6% (95% CI: 86.89-97.93%), and an overall accuracy of 97.8% (95% CI: 94.43-99.06%). No major complications were observed. Round shape and heterogeneous echogenicity were independently associated with LN malignancy. Overall survival was significantly shorter in patients with mediastinal LN metastases than in those without (log rank test, p = 0.008).

**Conclusions:**

The EBUS-TBNA procedure is a safe, highly accurate tool for evaluating thoracic lymphadenopathy in patients with an ETM. Its findings enable reliable identification of malignant involvement and may support clinical decision-making. Sonographic features may further assist in optimizing LN selection.

## INTRODUCTION

Radiological detection of mediastinal and hilar lymph node (LN) involvement represents a diagnostic challenge. Such involvement is relatively common in patients with extrathoracic malignancies (ETMs), occurring in approximately 30% of cases. When confirmed as metastatic disease, it typically signifies an advanced tumor stage and correlates with a significantly worse prognosis.^1^ ^-^ ^4^ Therefore, determining the underlying cause of mediastinal lymphadenopathy in patients with an ETM is a clinical imperative, because it may prevent the administration of inappropriate cytotoxic therapies and allow the implementation of targeted treatment strategies tailored to the specific etiology.^5^

Among the available diagnostic modalities, EBUS-TBNA is an established first-line, minimally invasive technique for evaluating mediastinal and hilar LNs with alterations of uncertain origin. Over the past two decades, EBUS-TBNA has been widely standardized and adopted, particularly in the diagnosis and staging of non-small cell lung cancer (NSCLC) as well as in the investigation of various intrathoracic abnormalities, including parenchymal lesions.^6^ ^,^ ^7^ The EBUS-TBNA modality, which allows diagnostic and staging procedures to be performed simultaneously through real-time acquisition of cytological samples, has an excellent safety profile and a low rate of complications.^8^ ^-^ ^10^

The reported sensitivity of EBUS-TBNA for detecting LN metastases from an ETM ranges from 85% to 90%, although it may decrease to approximately 55% in cases of occult metastases that are not radiologically evident on imaging.^1^ The most common ETMs leading to mediastinal nodal metastases include head and neck tumors, as well as colorectal, breast, and prostate carcinomas, all characterized by high recurrence rates and the need for accurate thoracic evaluation.^10^ ^,^ ^11^

Despite its established role in thoracic malignancies and nonmalignant diseases such as NSCLC, sarcoidosis, and tuberculosis, the diagnostic performance of EBUS-TBNA in mediastinal metastases from an ETM remains unclear. The literature is limited by heterogeneous study designs and small sample sizes, as well as by considerable variability in diagnostic accuracy and follow-up duration across studies.^2^ ^-^ ^4^ ^,^ ^12^ ^-^ ^22^ In addition, it has been hypothesized that early detection of mediastinal involvement has adverse prognostic implications.^23^

The objective of this study was to provide contemporary, real-world evidence on the performance of EBUS-TBNA in patients with an ETM and suspected mediastinal or hilar LN involvement, based on the evaluation of a large consecutive cohort at a well-established interventional pulmonology program with long-term follow-up. We integrated diagnostic performance, sonographic features, and exploratory survival analyses, in order to better reflect the role of EBUS-TBNA in routine multidisciplinary decision-making for patients with this complex condition.

## METHODS

### Study design and setting

This was an ambispective observational study conducted at the interventional pulmonology program of a tertiary care hospital. We evaluated a cohort of consecutive patients with a previous or current diagnosis of ETM and suspected thoracic lymphadenopathy who underwent EBUS-TBNA between January of 2012 and December of 2022. No formal sample size calculation was performed; all consecutive patients who met the inclusion criteria were included. Patients in whom there were medical contraindications to bronchoscopy were excluded. The study protocol was approved by the local institutional ethics committee, and all procedures were conducted in accordance with the ethical principles of the Declaration of Helsinki. All participating patients gave written informed consent.

As part of the oncological evaluation, all patients underwent CT, ^18^F-FDG PET/CT, or both. The use of EBUS-TBNA was indicated in the presence of LNs ≥ 10 mm in the short axis on CT or hypermetabolic activity on ^18^F-FDG PET/CT with a standardized maximum uptake value (SUV_max_) ≥ 2.5. These findings guided LN selection for EBUS-TBNA sampling, prioritizing accessible and highly suspicious nodes on the basis of their size, metabolic activity, and anatomical location (targeted exploration).

The EBUS-TBNA procedures were performed by experienced interventional pulmonologists who had access to the patient clinical histories and imaging findings, including ^18^F-FDG PET/CT data when available. The samples were interpreted by cytopathologists who were blinded to the final referral diagnosis but were aware of the EBUS-TBNA findings. 

Endobronchial ultrasound was performed with a linear-array ultrasound video bronchoscope (EB-1970UK 2.0; Pentax Medical, Tokyo, Japan) and 22-gauge needles (ECHO-HD-22-EBUS-P; Cook Medical, Rockville, MD, USA). The EBUS-TBNA procedure was performed under conscious sedation with individualized doses of intravenous propofol, midazolam, and fentanyl, or under general anesthesia with ventilatory support as needed under anesthesiologist control. A minimum of three punctures of at least one of the identified abnormal LNs were performed. A cytopathologist was present for rapid on-site evaluation, to determine sample adequacy. The material obtained was processed as direct smears, needle washings, or cell blocks. 

The samples were prepared per standard protocols. Immunocytochemistry and molecular testing were performed in selected cases. Cytological adequacy was defined as the presence of mature lymphocytes or diagnostic cellular components. The cytological samples obtained were classified as follows: neoplastic, when definitive features of malignancy were identified; benign, when cytology showed features of a specific benign or nonmalignant condition, such as granulomatous inflammation or infectious disease, supported by the clinical, radiological, and microbiological context when appropriate; or nondiagnostic, when only lymphocytes were observed in significant numbers, consistent with normal LNs. 

Demographic, clinical, imaging, procedural, and histological data were collected. The EBUS-TBNA results were classified according to predefined diagnostic criteria, and a composite reference standard was used. Benign or nonmalignant EBUS-TBNA results were classified as true negative (TN) when benignity/nonmalignancy was confirmed by surgical biopsy or stability/regression of lymphadenopathy during long-term clinical and radiological follow-up (for ≥ 24 months). In routine clinical practice, additional invasive procedures were not systematically performed after a negative EBUS-TBNA result unless persistent clinical or radiological suspicion of malignancy remained, or further confirmation was recommended by the multidisciplinary board. Therefore, long-term follow-up was accepted as part of the reference standard in patients without surgical confirmation.

Malignant cytology was considered definitive evidence of malignancy and was classified as a true-positive result, including metastatic disease, lymphoma, and primary lung cancer. For the diagnostic accuracy analysis, cases in which EBUS-TBNA demonstrated malignancy were considered true positives regardless of whether complete tumor subtyping or identification of the primary site was subsequently achieved.

False-negative (FN) cases were defined as those with an initially negative EBUS-TBNA result followed by a confirmed malignant diagnosis through an alternative invasive procedure or documented radiological progression. In nondiagnostic cases, patients were followed clinically and radiologically for a minimum of 24 months or alternative diagnostic tests were conducted, according to clinical judgment. 

The definitions were applied consistently across all cases to ensure a uniform classification of diagnostic outcomes. When EBUS-TBNA had already established malignancy, no subsequent invasive procedures for additional histological characterization of tumor subtyping were employed to modify diagnostic accuracy estimates. Malignant cytology was considered definitive evidence of disease in this oncological setting; therefore, no cases were classified as false positives. Consequently, specificity and positive predictive value were not calculated, and diagnostic performance was assessed using standard formulas for sensitivity, negative predictive value (NPV), and overall accuracy. Insufficient or nondiagnostic samples were excluded from the calculation of ETM-specific prevalence.

### Statistical analysis

Statistical analysis was performed with IBM SPSS Statistics software package, version 25.0 (IBM Corp., Armonk, NY, USA). Normally distributed continuous variables (assessed via the Kolmogorov-Smirnov or Shapiro-Wilk test) are expressed as mean and standard deviation, asymmetrically distributed variables are expressed as median and interquartile range, and categorical variables are expressed as frequencies and percentages. Comparisons of means were calculated with Student’s t-test or ANOVA if the distribution was normal or parametric, whereas they were calculated with the Mann-Whitney U test if the distribution was asymmetrical. Values of p < 0.05 were considered statistically significant.

Overall survival (OS) was defined as the time elapsed from the diagnosis of mediastinal involvement or previously diagnosed ETM to death from any cause. Kaplan-Meier curves with log-rank test and Cox proportional hazards model were employed to assess OS for survival analysis. All of the patients included had complete clinical, imaging, and procedural datasets; patients with insufficient follow-up for survival assessments were excluded from survival analyses.

## RESULTS

### Lymph node characteristics and diagnostic accuracy

We included a total of 186 consecutive patients with suspected or confirmed mediastinal or hilar LN metastases from an ETM, identified on the basis of the results of EBUS-TBNA or other complementary diagnostic procedures. Of those 186 patients, 176 (94.6%) had a known history of malignancy. All of the patients had undergone CT, ^18^F-FDG PET/CT, or both within 2 months before the procedure, and an ^18^F-FDG PET/CT scan was available before EBUS-TBNA in 124 cases (66.7%). 

Table 1 summarizes the main clinical and procedural characteristics of the cohort, as well as the distribution of the ETM types identified. Cases with an FN result (confirmed by surgery or oncologic follow-up) were excluded from this table, as were those with a nondiagnostic EBUS-TBNA result, as well as four cases with unknown primary tumors (three diagnosed by EBUS-TBNA and one classified as an FN result by clinical follow-up). Valid material for diagnostic analysis was obtained in 180 (96.8%) of the 186 procedures.

**Table 1:** General characteristics of the study population and technique.

Variable	(N = 186)
Age (years), mean ± SD	65 ± 9.78
Sex, n (%)	
Male	121 (65.1)
Female	65 (34.9)
Smoking status, n (%)	
Current smoker	37 (19.9)
Former smoker	66 (35.5)
Never smoker	83 (44.6)
setting, n (%)	
In-hospital	18 (9.7)
Outpatient	168 (90.3)
Sedation, n (%)	
Conscious/deep sedation	184 (98.9)
General anesthesia	2 (1.1)
Respiratory support during the procedure	
Conventional oxygen therapy	180 (96.8)
Non-invasive ventilation	4 (2.1)
Invasive mechanical ventilation	2 (1.1)
Rapid on-site evaluation, n (%)	176 (94.6)
ETM type, n (%)	
Gastrointestinal	21 (22.6)
Urological	21 (22.6)
Breast	19 (20.4)
Head and neck	14 (15.1)
Melanoma	6 (6.4)
Other*	9 (9.7)

EBUS-TBNA: endobronchial ultrasound-guided transbronchial needle aspiration. *Thyroid, pancreatic, esophageal, thymus, liver, bone, and neuroendocrine.

A total of 237 LNs and 12 parenchymal lesions, all suspected of being malignant, were sampled by EBUS-TBNA. Each site received a median of three needle passes. Table 2 details the radiological and sonographic features of the LNs and lesions sampled, as well as their distribution by station.

**Table 2:** Imaging characteristics of the lymph nodes and anatomic mediastinal regions explored by endobronchial ultrasound-guided transbronchial needle aspiration.

Characteristic	(N = 186)
Lymph node (station), n (%)	
High right paratracheal (2R)	10 (5.2)
High left paratracheal (2L)	4 (2.1)
Low right paratracheal (4R)	55 (28.6)
Low left paratracheal (4L)	6 (3.1)
Subcarinal (7)	63 (32.8)
Right hilar (10R)	13 (6.8)
Left hilar (10L)	5 (2.6)
Right interlobar (11R)	24 (12.5)
Left interlobar (11L)	12 (6.3)
Size (cm), mean ± SD	
Lymph node	1.3 ± 0.7
Parenchymal lesions	1.5 ± 0.7
^18^F-FDG PET/CT uptake (SUV_max_), median (IQR)	
Lymph node	5.3 (3.6-8.9)
Positive by EBUS-TBNA	7.9 (4.2-11.8)
Negative by EBUS	4.5 (1.7-9.2)
Parenchymal lesion	6.5 (2.5-10)

EBUS-TBNA: endobronchial ultrasound-guided transbronchial needle aspiration; and SUV_max_: maximum standardized uptake value.

In comparison with LNs affected by benign or nonmalignant pathologies, those with metastases from an ETM were significantly larger in their short-axis diameter (1.9 cm vs. 1.4 cm; p = 0.001) and exhibited higher metabolic activity (SUV_max_ of 7.9 vs. 4.5; p = 0.001). Sonographic features of the LNs sampled were analyzed in relation to diagnostic outcomes (Table 3). Multivariate logistic regression identified round shape and heterogeneous echogenicity as independent predictors of malignancy.

**Table 3:** Sonographic characteristics of lymph nodes sampled by endobronchial ultrasound-guided transbronchial needle aspiration.

Characteristic	EBUS-TBNA		p-value		Adjusted OR (95% CI)*
	Positive	Negative			
	n (%)	n (%)	Univariate	Multivariate	
Shape					
Oval	13 (13.7)	53 (75.7)			
Round	82 (86.3)	17 (24.3)	< 0.001	0.001	6.3 (2.31-16.93)
Margins					
Indistinct	26 (24.5)	57 (77)			
Distinct	80 (75.5)	17 (23)	< 0.001	0.056	2.8 (0.55-5.08)
Echogenicity					
Homogeneous	11 (10.4)	60 (81.1)			
Heterogeneous	95 (89.6)	14 (18.9)	< 0.001	0.001	16.8 (6.17-45.76)
Matting					
Absent	95 (89.6)	70 (94.6)			
Present	11 (10.4)	4 (5.4)	0.235	-	-
Central hilar structure					
Absent	102 (96.2)	52 (70.3)			
Present	4 (3.8)	22 (29.7)	0.004	0.077	4.3 (0.86-21.32)
Calcification					
Absent	104 (98.1)	69 (93.2)			
Present	2 (1.9)	5 (6.8)	0.096	-	-
Necrosis sign					
Absent	73 (68.9)	69 (93.2)			
Present	33 (31.1)	5 (6.8)	< 0.001	0.094	2.7 (0.34-4.96)

EBUS-TBNA: endobronchial ultrasound-guided transbronchial needle aspiration. *For malignancy.

Figure 1 shows a flow chart of the diagnostic outcomes and the reference standards used in order to confirm the final diagnosis. Overall, 90 of 176 patients (51.1%) showed evidence of tumor recurrence.

**Figure 1:**
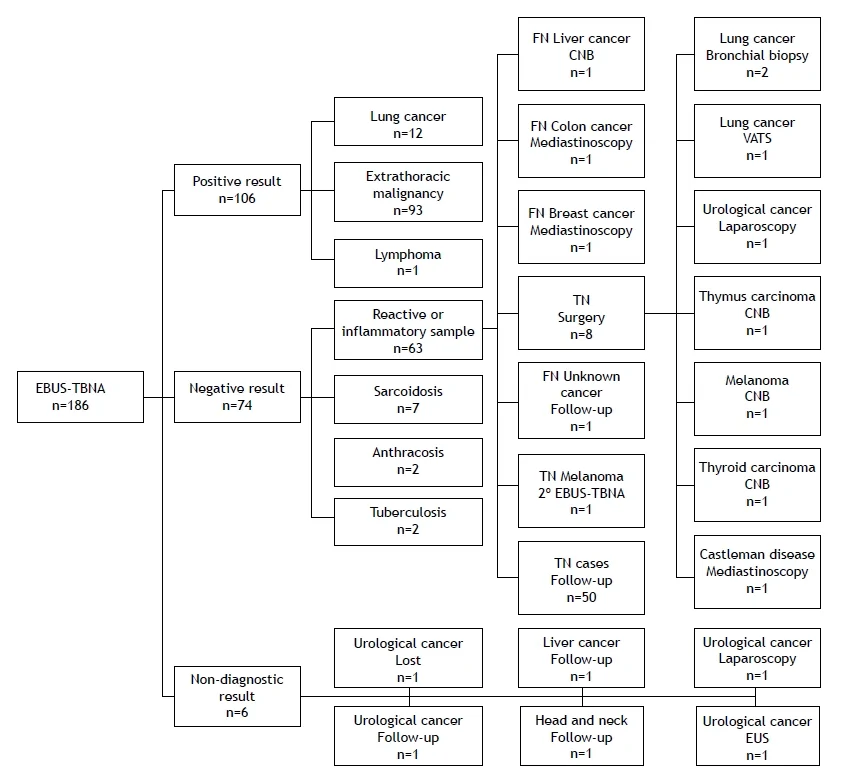
Flow chart of patient inclusion and final diagnostic classification according to cytological findings, surgical confirmation, or clinical-radiological follow-up. EBUS-TBNA, endobronchial ultrasound-guided transbronchial needle aspiration; EUS, endoscopic ultrasound; FN, false negative; TN, true negative; CNB, core needle biopsy; VATS, video-assisted thoracoscopic surgery.

Of the 186 patients evaluated, 106 (57%) were diagnosed with malignancy, and metastases from an ETM were confirmed in 93 of those. For statistical purposes, three patients diagnosed with metastatic disease from an unknown primary tumor were classified as having an ETM: one had a history of skin cancer, and two had no prior malignancy, one dying shortly after being diagnosed. Eleven patients (5.9%) were diagnosed with nonmalignant conditions (two with tuberculosis, seven with sarcoidosis, and two with anthracosis). In 63 patients (33.9%), EBUS-TBNA was nondiagnostic for malignancy or an alternative nonmalignant diagnosis. Of those 63 patients, 12 (6.5%) underwent additional invasive diagnostic procedures following a multidisciplinary tumor board review, whereas the remaining cases were managed with clinical and radiological follow-up. In addition, two patients from the nondiagnostic group required further invasive evaluation, resulting in a total of 14 patients undergoing complementary procedures. Of those 14 patients, four were referred for mediastinoscopy. In two of those four patients, mediastinoscopy yielded positive results for LNs previously been classified as negative on EBUS-TBNA, and the EBUS-TBNA findings were therefore considered FN results on histological examination. In the 10 remaining cases, complementary invasive procedures were performed to confirm staging or exclude malignancy in the context of high clinical suspicion and yielded negative results. In addition, one patient with no history of cancer had a negative EBUS-TBNA result and was diagnosed with Castleman’s disease on mediastinoscopy; in that case, the EBUS-TBNA finding was classified as a TN result. Three patients with negative EBUS-TBNA findings were later diagnosed with primary lung cancer via bronchial biopsy or lobectomy (by video-assisted thoracoscopic surgery). Because there was no apparent mediastinal involvement (N0 stage), the EBUS-TBNA findings were classified as TN results with respect to mediastinal staging in all three cases. Data for the remaining patients corresponded to alternative diagnostic procedures or follow-up strategies, as detailed in Figure 1. 

The remaining patients with inconclusive EBUS-TBNA findings were followed for at least 24 months with clinical and radiological examinations. In four cases (2.2%), the findings were classified as FN results: in three, metastases (from colorectal, breast, and liver cancer) were confirmed by surgical biopsy; in the remaining case, despite the lack of histological confirmation, the findings were classified as indicative of metastasis on the basis of the clinical course (rapid progression and death within 2 months) and history of head and neck cancer.

Twelve patients were diagnosed with lung cancer, and two of those patients experienced recurrences. The EBUS-TBNA procedure successfully identified the histologic subtype (adenocarcinoma and large-cell carcinoma), and in all cases, provided material adequate for molecular analyses.

A second primary malignancy was identified in eight patients (4.6%), including seven patients with lung cancer and one with urological cancer, all of whom had a history of cancer. In a separate subgroup, six patients without a history of cancer were diagnosed with a previously unsuspected primary tumor following EBUS-TBNA, including one lymphoma, two adenocarcinomas of unknown origin, and three urological cancers. Although specific treatment changes were not systematically recorded, EBUS-TBNA results were considered to have a direct impact on clinical management. The overall prevalence of malignancy in the cohort was 59.1% (identified in 110 of the 186 patients), including metastatic ETMs, primary lung cancer, and lymphoma.

The sensitivity of EBUS-TBNA for malignancy was 96.4% (95% CI: 90.98-98.62%), with an NPV of 94.6% (95% CI: 86.89-97.93%) and an overall accuracy of 97.8% (95% CI: 94.43-99.06%). No major complications were observed following the procedure. 

## PROGNOSTIC ANALYSIS

Three patients (1.6%)-two with valid samples receiving a diagnosis of breast LN metastases and one with a nondiagnostic sample-were lost to follow-up within the first 12 months. Because no conclusive follow-up data were available in the medical records, those patients were excluded from the survival analyses. The median OS in the study cohort was 32 months (IQR, 13-104 months). Figure 2A illustrates the OS according to the EBUS-TBNA diagnostic results, while Figure 2B shows the OS based on the type of ETM. 

**Figure 2:**
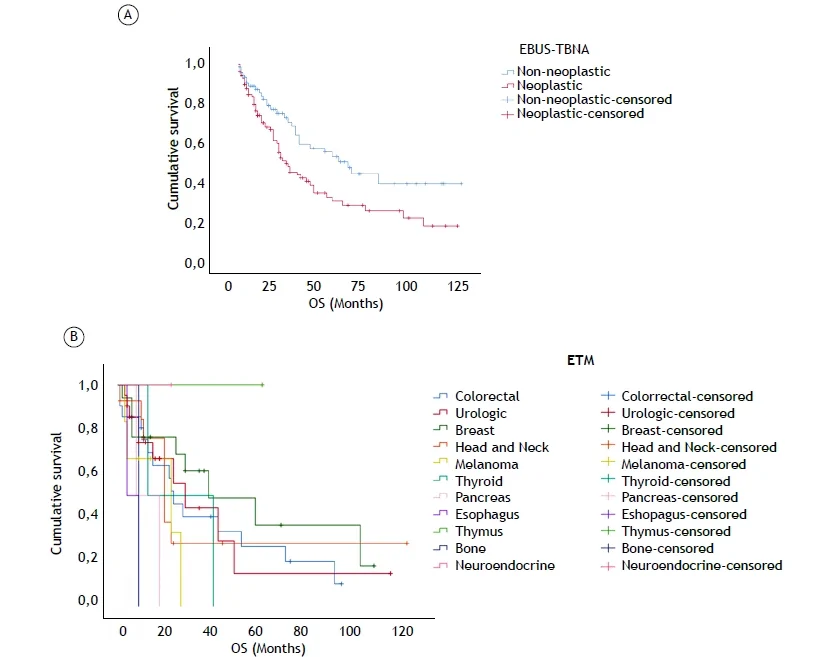
Kaplan-Meier analyses of overall survival (OS). A: OS according to endobronchial ultrasound-guided transbronchial needle aspiration (EBUS-TBNA) diagnostic result (neoplastic or non-neoplastic; log-rank test, p = 0.008). B: OS stratified by type of extrathoracic malignancy (ETM, log-rank test, p = 0.038).

Patients with LN metastases diagnosed by EBUS-TBNA had significantly shorter median survival than did those with negative results-24 months (95% CI: 19-29 months) versus 57 months (95% CI: 28-86 months); log-rank test, p = 0.008. Two ETM subtypes (thymic carcinoma and multiple endocrine neoplasia) were excluded from the final analysis because of 100% censoring. 

Cumulative survival analysis showed a median OS of 24 months in the patients diagnosed with an ETM. Those with breast, urologic, or colorectal malignancies exhibited the longest survival, with median OS of 39, 29, and 24 months, respectively. In contrast, those with esophageal, thyroid, or pancreatic malignancies showed 100% mortality and the shortest survival times (4, 13, and 8 months, respectively). 

A Cox proportional hazards model was used in order to assess variables associated with OS. In this model, melanoma, head and neck cancer, colorectal cancer, and malignancies in the “other ETMs” group (thyroid, pancreatic, esophageal, and bone cancer) were associated with an increased risk of death. Melanoma showed the strongest association with mortality (HR = 4.60; 95% CI: 1.48-14.31; p = 0.008), followed by head and neck cancer (HR = 3.07; 95% CI: 1.24-7.55; p = 0.015), malignancies in the “other ETMs” group (HR = 3.07; 95% CI: 1.03-9.19; p = 0.045) and colorectal cancer (HR = 2.89; 95% CI: 1.36-6.17; p = 0.006). Breast cancer showed a nonsignificant trend toward increased mortality (HR = 2.48; 95% CI: 0.94-6.53; p = 0.065). Additional variables that demonstrated nonindependent but suggestive associations with increased risk in patients with an ETM included male sex (HR = 2.02; 95% CI: 0.96-4.27; p = 0.067), a high smoking index (smoking exposure > 20 pack-years; HR = 1.85; 95% CI: 0.88-3.86; p = 0.104), and hypertension (HR = 1.15; 95% CI: 0.93-1.42; p = 0.212).

## DISCUSSION

Mediastinal or hilar LN involvement in patients with an ETM constitutes a clinically relevant finding, usually associated with advanced disease and an unfavorable prognosis. In this scenario, accurate evaluation and tissue confirmation of nodal involvement are essential to guide treatment planning and prognostic assessment.^1^ ^,^ ^2^ The diagnostic role of EBUS-TBNA in this population has previously been evaluated in several studies and systematic reviews. In the present study, we provide a real-world assessment of EBUS-TBNA in this setting, based on a large cohort with long-term follow-up. By integrating diagnostic performance, sonographic features associated with malignancy, and survival outcomes, our findings offer a comprehensive characterization of its clinical role in routine practice, in which the procedure is intended not only to confirm malignancy but also to guide subsequent diagnostic, staging, restaging, and treatment decisions.

In our cohort, EBUS-TBNA demonstrated high diagnostic performance, with a sensitivity of 96.4%, an NPV of 94.6% and an overall accuracy of 97.8%, consistent with previously reported series (Table 4). The rates of FN results (2.2%) and inadequate or insufficient samples (3.2%) were within acceptable ranges for clinical practice, as defined by the World Association for Bronchology and Interventional Pulmonology.^24^ Although not all negative cases received surgical confirmation-particularly when clinical, radiological, and multidisciplinary assessment supported surveillance-long-term clinical and radiological follow-up (≥ 24 months) was used as part of the reference standard. This approach reflects real-world clinical decision-making but introduces potential verification bias, given that a small number of occult FN cases may have been misclassified as TN cases. Consequently, sensitivity and the NPV may be overestimated and should be interpreted within this diagnostic framework.

**Table 4:** Summary of published studies on the diagnostic accuracy of endobronchial ultrasound-guided transbronchial needle aspiration for mediastinal and hilar lymph node metastases from extrathoracic malignancies.

Study	Year	Country	n	Sensitivity	Specificity	NPV	Accuracy
				(%)	(%)	(%)	(%)
Song et al.^12^	2011	Korea	56	88	-	85	93
Park et al.^13^	2011	Korea	59	81	100	-	-
Navani et al.^3^	2011	England	161	87	-	73	88
Tournoy et al.^16^	2011	Belgium	92	85	-	76	-
Parmaksiz et al.^14^	2012	Türkiye	48	89.2	100	86.9	93.7
Ozgül et al.^17^	2013	Türkiye	40	90	100	90.9	95
Sanz-Santos et al.^15^	2013	Spain	117	86.4	-	75	90.3
Şentürk et al.^27^	2014	Türkiye	54	93	100	92.6	96.3
Erer et al.^19^	2016	Türkiye	63	78.2	100	88.3	91.8
Dziedzic et al.^2^	2016	Poland	32	93.3	100	87.5	78.8
Tertemiz et al.^4^	2017	Türkiye	91	90.2	100	92.5	-
Guarize et al.^20^	2017	Italy	176	95.7	100	92.3	97.2
Fournier et al.^21^	2019	France	185	68.4	100	53.3	54.6
Demirdöğen et al.^22^	2020	Türkiye	109	76.2	100	94.6	95.4
Luthra et al.^29^	2021	India	57	90	-	90	94.7
Martin-Deleon et al.^23^	2022	Spain	161	88	100	87	-
Sana et al.^28^	2024	Pakistan	216	90.9	-	-	96.9
This study	2025	Spain	186	96.4	-	94.6	97.8

NPV: negative predictive value.

Recent methodological recommendations for advanced diagnostic bronchoscopy have emphasized the need for strict, transparent definitions of diagnostic yield, particularly in studies evaluating peripheral lung lesions (PLLs).^25^ ^,^ ^26^ In that setting, clinical or radiological follow-up may be incorporated into the reference standard to determine the final benign or malignant nature of the target lesion, although it should not be used in order to reclassify nonspecific or nondiagnostic bronchoscopic findings as diagnostic results. These recommendations provide a valuable framework for improving the quality and comparability of bronchoscopy studies; however, their primary scope is PLLs, which differs from nodal assessment by EBUS-TBNA. Unlike peripheral lung nodules, mediastinal and hilar LNs sampled by EBUS-TBNA are usually anatomically well-defined targets, and cytological confirmation of malignancy is considered definitive in oncological practice. In addition, no specific consensus currently requires the strict diagnostic-yield definition proposed for PLLs to be applied unchanged to EBUS-TBNA studies of thoracic lymphadenopathy. Therefore, we assessed diagnostic accuracy using predefined criteria and a transparent composite reference standard, while explicitly acknowledging the potential for verification bias in negative cases.

Mediastinal LN metastases from ETM were identified in 52.2% of patients, a prevalence comparable to that reported in recent studies.^15^ ^,^ ^23^ ^,^ ^27^ ^,^ ^28^ The nodal stations most commonly involved were the subcarinal station (station 7) and the right paratracheal station (station 4R), a finding also in line with those of previous studies.^20^ ^,^ ^28^ ^,^ ^29^ More than half of the patients with a history of cancer presented with disease recurrence, particularly those with breast, melanoma, or head and neck cancers. In contrast, genitourinary malignancies (although frequently described in the literature as having a high potential for thoracic dissemination) showed a lower recurrence rate in our cohort, which may reflect differences in tumor biology, improvements in screening programs, differences in referral pathways, and earlier oncological management.^30^

An additional relevant finding was the diagnostic contribution of EBUS-TBNA in patients without a history of cancer. In six cases, the procedure enabled the identification of previously unsuspected malignancies, highlighting its value in selected clinical scenarios. This underscores the practical value of EBUS-TBNA in the differential diagnosis of mediastinal and hilar lymphadenopathy beyond simple confirmation of recurrence. In addition, the combination of rapid on-site evaluation, cell block preparation, and immunohistochemistry contributed to the high diagnostic yield and allowed molecular testing and adequate neoplasm characterization in selected patients.^31^ ^-^ ^34^ However, additional studies on metastases to LNs from an ETM are required in order to confirm the extrapolation of these results.

Imaging and certain sonographic features also demonstrate a consistent association with malignancy. Metastatic LNs showed higher SUV_max_ values on ^18^F-FDG PET/CT and larger nodal size, supporting the role of CT and ^18^F-FDG PET/CT in preprocedural stratification and target selection.^35^ In addition, round shape and heterogeneous echogenicity were independently associated with malignancy, although no single ultrasound feature was sufficiently sensitive or specific, which is consistent with the findings of previous studies.^7^ ^,^ ^35^ These findings support a multimodal approach integrating clinical, imaging, and sonographic data to improve the diagnostic approach.^24^ ^,^ ^36^

From a survival perspective, the median OS was significantly shorter in patients with EBUS-TBNA-confirmed mediastinal or hilar metastases than in those without nodal involvement (24 vs. 57 months; log-rank p = 0.008). This finding is clinically coherent, because malignant involvement of thoracic LNs detected by EBUS-TBNA in patients with an ETM usually reflects systemic progression, recurrence, or advanced tumor burden. 

Survival outcomes varied according to ETM subtype, highlighting the marked biological and clinical heterogeneity of this population, with poorer outcomes observed in patients with metastases from colorectal cancer, melanoma, and head and neck cancer, all of whom presented a median survival of less than 24 months and five-year survival rates under 30%.^37^ ^-^ ^39^ In contrast, breast and urological cancers were associated with more favorable survival. Our findings differ somewhat from those of DeLeon et al.,^23^ who found a worse prognosis to be worse for patients with metastases from breast or gynecological cancer.^23^ In the Cox regression analyses, melanoma, head and neck cancer, colorectal cancer and “other ETMs” (a pooled category including thyroid, esophageal, pancreatic, and bone cancers) were associated with higher mortality. These findings are plausible from an oncological perspective, but they may also reflect differences in patient populations, tumor biology, treatment exposure, referral patterns, and study design; therefore, they should be interpreted as exploratory.

The present study had several limitations. Its ambispective single-center design may have introduced selection bias and limits the ability to control for unmeasured confounding, although the inclusion of consecutive patients from a well-established interventional pulmonology program provides procedural consistency and reflects routine clinical practice. In addition, the absence of systematic surgical confirmation after negative EBUS-TBNA results may have led to overestimation of sensitivity and the NPV, despite the use of prolonged clinical-radiological follow-up as part of a composite reference standard. Furthermore, the survival analyses should be interpreted as exploratory, including treatment strategies, tumor burden, disease stage, molecular profile, performance status, and subsequent therapies, none of which were fully captured or adjusted for. Moreover, the heterogeneity of ETM subtypes and the limited number of patients within each tumor category also precluded robust subgroup-specific survival estimates. Finally, ultrasound assessment, even when performed by experienced bronchoscopists, as it was in the present study, is partly operator-dependent, and interobserver variability was not formally assessed. 

Despite the limitations, this study provides contemporary real-world evidence supporting EBUS-TBNA as a minimally invasive front-line procedure for suspected mediastinal or hilar LN involvement in patients with an ETM. Its main contribution is the integrated assessment of diagnostic accuracy, radiological findings, sonographic features, cytological characterization, and exploratory survival outcomes within a large, consecutive single-center cohort with long-term follow-up. The EBUS-TBNA results showed high diagnostic accuracy, whereas sonographic features such as round shape and heterogeneous echogenicity were associated with malignancy and may support pre-procedural assessment. Malignant nodal involvement was associated with shorter survival, most likely reflecting the burden of underlying disease. In multidisciplinary practice, cytological confirmation by EBUS-TBNA may inform staging, restaging, treatment intent, and therapeutic planning. Future prospective multicenter studies with standardized reference standards and adequately powered tumor-specific analyses are warranted. Emerging sampling strategies, such as EBUS-guided transbronchial mediastinal cryobiopsy, may further improve tissue acquisition in selected cases, particularly when cytology is inconclusive or when larger samples are required for comprehensive immunohistochemical or molecular characterization.^40^
